# Sepsis induced cognitive impairments by disrupting hippocampal parvalbumin interneuron-mediated inhibitory network via a D4-receptor mechanism

**DOI:** 10.18632/aging.102755

**Published:** 2020-02-04

**Authors:** Muhuo Ji, Shuming Li, Ling Zhang, Yuzhu Gao, Qiuting Zeng, Minjie Mao, Jianjun Yang

**Affiliations:** 1 Department of Anesthesiology, Pain and Perioperative Medicine, The First Affiliated Hospital of Zhengzhou University, Nanjing, China; 2 Department of Anesthesiology, Zhongda Hospital, Medical School, Southeast University, Nanjing, China; 3 Department of Anesthesiology, Jinling Hospital, School of Medicine, Nanjing University, Nanjing, China

**Keywords:** cognitive impairment, D4 receptor, parvalbumin, sepsis, γ oscillation

## Abstract

Patients who suffer sepsis often develop cognitive impairments, yet the underlying mechanisms largely remain to be elucidated. Increasing evidence has suggested that parvalbumin (PV) interneurons are required for the synchronization of neural activities and higher brain processes, whereas its dysfunction is implicated in many psychiatric disorders. In the present study, we examined the role of hippocampal PV interneuron-mediated inhibitory network in a rat model of polymicrobial sepsis induced by cecal ligation and puncture (CLP) and also explored the underlying mechanism. Here we showed that CLP-induced cognitive impairments, which were accompanied by significantly decreased expressions of PV and dopamine 4 (D4) receptor, decreased slow γ oscillation band, and reduced frequency of miniature inhibitory postsynaptic currents (mIPSCs). Notably, D4 receptor agonist RO-10-5824 treatment was able to reverse most of these abnormities. In summary, our study suggests that sepsis might disrupt PV interneuron-mediated network function that is dependent on the D4 receptor, leading to abnormal γ oscillation and consequent cognitive impairments.

## INTRODUCTION

Brain dysfunction is a common complication in patients with sepsis and is associated with increased mortality, decreased quality of life and long-term neurocognitive consequences [1–3]. It is clinically characterized by an acute alteration of consciousness, and less frequently by seizures or focal neurologic signs [3]. Importantly, sepsis survivors often present long-term cognitive impairment and some of these alterations resemble the pathophysiological mechanisms of neurodegenerative diseases [4]. In preclinical studies, it has been shown that sepsis induced by either lipopolysaccharide administration or cecal ligation and puncture (CLP) impairs learning and memory [5–8]. However, the precise mechanisms underlying sepsis-induced cognitive impairment remain largely to be elucidated.

Parvalbumin (PV) interneurons constitute a subpopulation of GABAergic neurons that control the output of principal neurons and are necessary for rhythmic local field potentials (LFP), facilitating information processing during cognitive tasks [9, 10]. Optogenetic activation of PV interneurons is sufficient to modulate γ oscillations, cortical circuit synchrony, and behaviors [10, 11]. By contrast, dysfunction of PV interneurons has been implicated in abnormal γ oscillations and cognitive impairments associated with many neuropsychiatric disorders, including Alzheimer's disease (AD) [12], schizophrenia [13], and major depression [14]. Consistently, our previous study has shown that selective phenotype loss of PV interneurons might contribute to cognitive impairments in an animal model of sepsis induced by CLP [6]. However, it remains unclear whether and how sepsis alters the activity of the PV interneuron-mediated inhibitory network of the hippocampus.

Several lines of evidence have demonstrated the potential role of the dopamine 4 (D4) receptor in the regulation of GABAergic transmission, γ oscillations, and potentially cognitive processes [15]. It has been reported that D4 receptor agonists may improve cognitive functions by modulating γ activity [16–18]. Given the key role of the PV interneuron-mediated network function, the present study thus hypothesized that sepsis impaired the structural and functional integrity of the hippocampal PV-mediated inhibitory network that is dependent on D4 receptor, leading to abnormal γ oscillations and consequent cognitive impairments.

## RESULTS

### Survival rate after surgery

**Figure 1:**
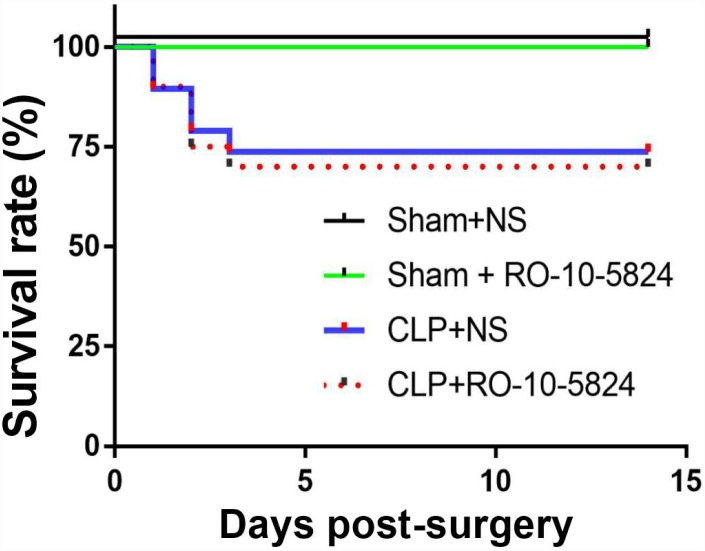
**Effects of CLP on survival rate.** CLP, cecal ligation and puncture. NS, normal saline.

To investigate the effects of CLP on survival rate, the rats were monitored for 14 days after surgery. No animal died in the sham groups. The survival rate in the CLP + NS and CLP+ RO-10-5824 group was 73.7% and 70.0%, respectively (*P* = 0.8116, n = 15-20, Figure 1). These results suggested that our experimental protocol induced a medium sepsis animal model.

### Decreased hippocampal PV expression and slow γ band power after CLP

**Figure 2:**
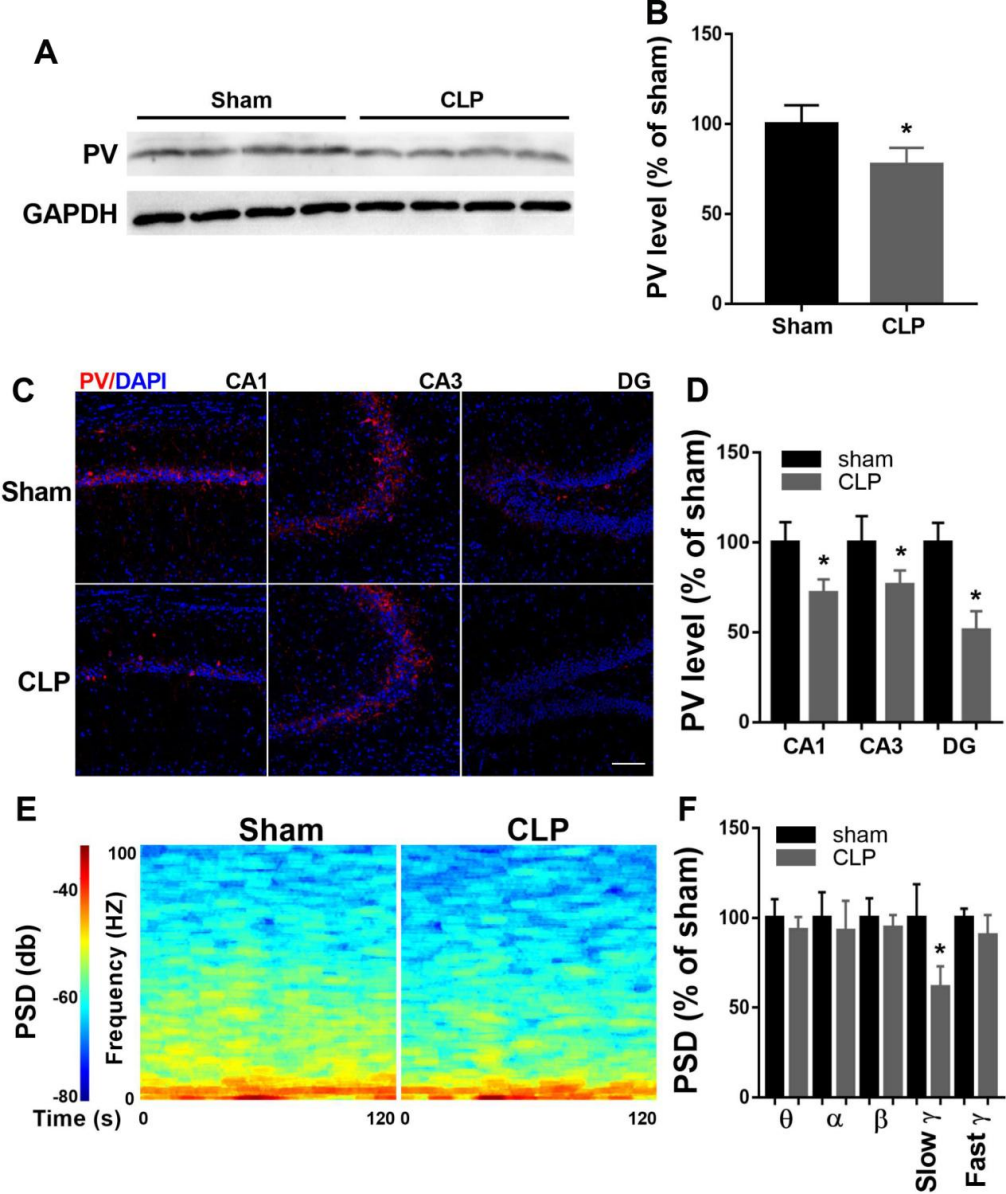
**Altered hippocampal PV expression and LFP after CLP.** (**A**–**B**) PV expression decreased significantly at 14 days after CLP compared with sham group (n = 4). (**C**–**D**) Decreased PV expression was observed in all subregions of the hippocampus 14 days after CLP (n = 4). (**E**–**F**) Slow γ oscillation band was significantly decreased in CLP group when compared with sham group, but there was no difference in θ, α, β, or fast γ band power between these two groups (n = 3). Data are shown as mean ± SD, *P < 0.05 vs sham group, scale bar = 50 μm.

To determine PV expression, we performed western blot of hippocampal protein extracts obtained from sham and CLP rats 14 days after surgery. As shown in Figure 2A–2B, PV expression decreased significantly at 14 day after CLP when compared with sham group (t = 2.862, *P* = 0.0287, n = 4). To further confirm this result, we performed immunofluorescence to detect PV expression. Similarly, decreased PV expression was observed in all subregions (CA1, CA3, DG) of the hippocampus 14 days after CLP (all *P* < 0.05; n = 4, Figure 2C–2D).

Since PV interneurons are crucial for cortical network function, we measured LFP of the CA1 14 days after surgery when the rats performed the novel object recognition test. As shown in Figure 2E–2F, slow γ oscillation band was significantly decreased in CLP group when compared with sham group (t = 3.489, *P* = 0.013, n = 4), but there was no difference in θ, α, β, or fast γ band power between these two groups (all *P* > 0.05).

### RO-10-5824 treatment reversed decreased GABAergic transmission in the CA1 after CLP

**Figure 3:**
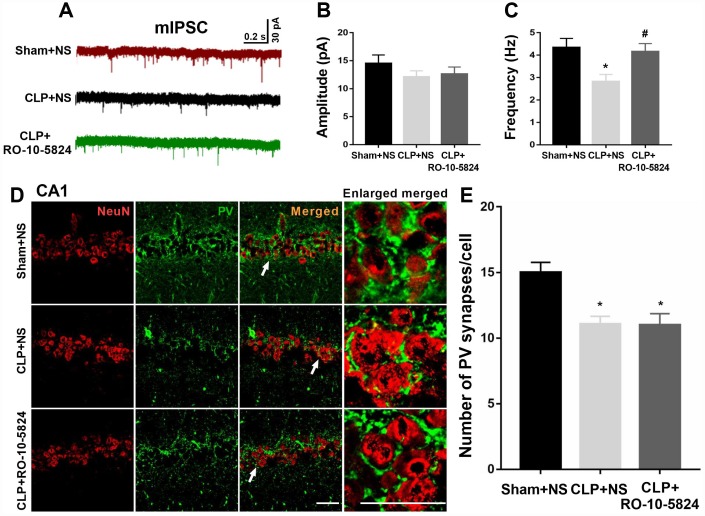
**RO-10-5824 treatment reversed decreased GABAergic transmission in the CA1 after CLP.** (**A**) Representative traces of mIPSCs in slices from sham + NS, CLP + NS, and CLP + RO-10-5824 rats. (**B**) CLP did not affect the amplitude of mIPSCs (n = 10 pyramidal cells from three rats in each group). (**C**) CLP induced a significantly decreased frequency of mIPSCs, which was prevented by RO-10-5824 treatment (n = 10 pyramidal cells from three rats in each group). (**D**-**E**) CLP significantly decreased the total number of PV synapses around the pyramidal neurons as compared with sham group, which was not affected by RO-10-5824 treatment (n = 15 pyramidal cells from three rats in each group). Data are shown as mean ± SD, *P < 0.05 vs sham + NS group; #*P* < 0.05 vs CLP + NS group, scale bar = 50 μm.

To determine whether sepsis impaired GABAergic inhibitory synaptic transmission in the CA1, we recorded mIPSCs from principal neurons 14 days after surgery. Although the amplitude of mIPSCs recorded from the CLP-exposed animals did not differ from that recorded in sham animals (F = 1.373, *P* = 0.2705, n = 4, Figure 3B), we found a significantly decreased frequency of mIPSCs (sham + NS: 4.3 ± 1.2 Hz; CLP + NS: 2.8 ± 1.0 Hz; CLP + RO-10-5824: 4.2 ± 1.1 Hz, F = 9.838, *P* = 0.0008, n = 4, Figure 3C), suggesting that these neurons received significantly decreased inhibitory drive than those in sham + NS group. RO-10-5824, a selective D4 receptor agonist normalized the mIPSC frequency in the CA1 after CLP.

Since PV interneurons mainly innervate the perisomatic domain of pyramidal neurons and plays a key role in generating network oscillations and cognition, we next measured a special type of inhibitory synapse named perisomatic synapses around the pyramidal neurons 14 days after surgery, which were NeuN-positive. We showed that CLP significantly decreased the total number of PV synapses around the pyramidal neurons as compared with sham + NS group (F = 5.245, *P* = 0.0129, n = 3, Figure 3D–3E), suggesting the loss of inhibitory synapses on pyramidal neurons. However, RO-10-5824 treatment was unable to increase the total number of PV synapses around the pyramidal neurons (Figure 3E).

### D4 receptor colocalizes in cells positive for PV and was decreased after CLP

**Figure 4:**
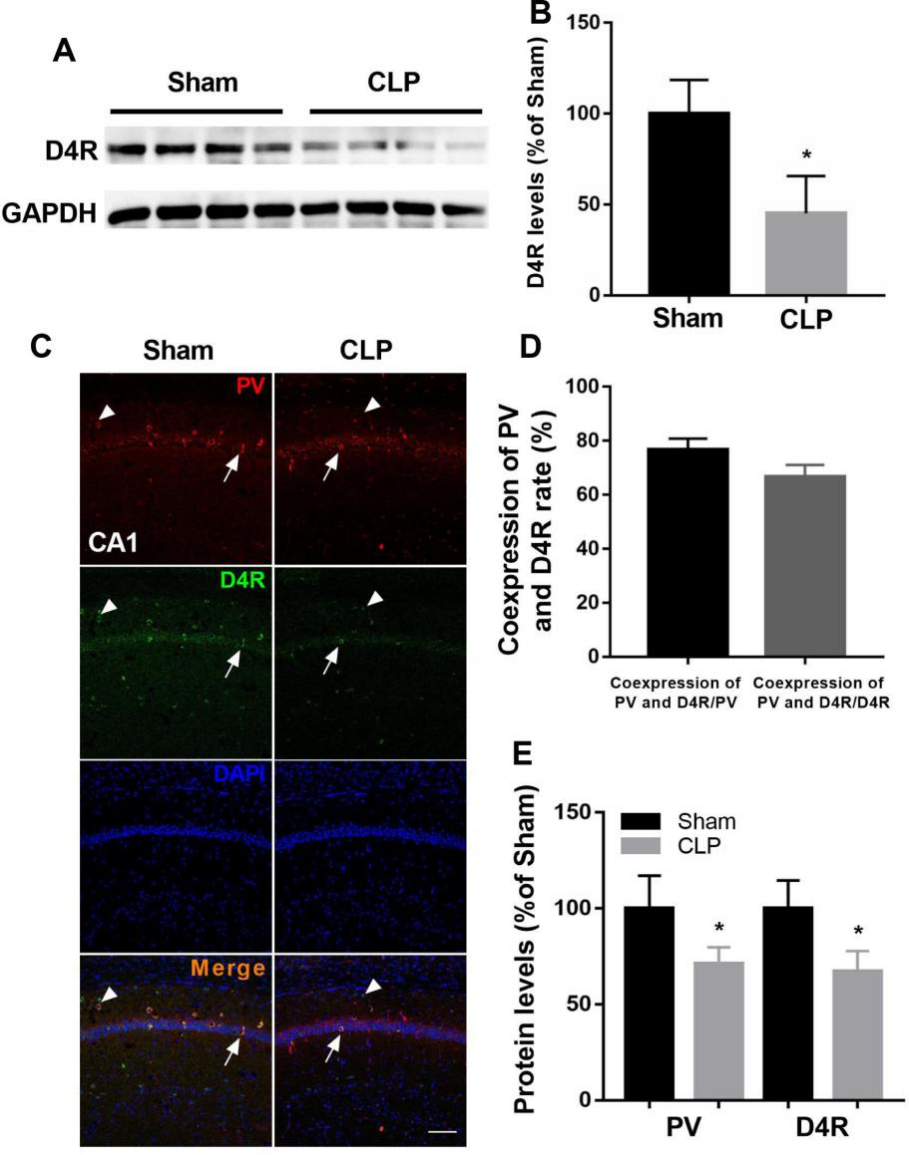
**D4 receptor colocalizes in cells positive for PV and was decreased after CLP.** (**A**–**B**) D4 receptor was significantly decreased after CLP. (**C**–**D**) Most PV interneurons co-express D4 receptor and a large proportion of D4 receptor-expressing neurons in the hippocampus co-express PV. Arrow: coexpress D4 receptor and PV; Arrowhead: express D4 receptor without parvalbumin. (**C** and **E**) Decreased PV and D4 expressions were observed after CLP when compared with sham + NS group. *P < 0.05 vs sham group, scale bar = 50 μm.

We showed D4 expression was significantly decreased following CLP by western blot analysis (t = 3.965, *P* = 0.0074; n = 4, Figure 4A–4B). We next employed double-immunohistochemistry using an antibody raised against the D4 receptor and PV in the CA1 region of the hippocampus 14 days after surgery. The results indicated that most PV interneurons co-express the D4 receptor and conversely a large proportion of D4 receptor-expressing neurons in the hippocampus co-express PV (Figure 4C–4D). In addition, we found significantly decreased PV and D4 expressions after CLP when compared with sham + NS group (PV: t = 3.369, *P* = 0.0098; D4R: t = 4.407, *P* = 0.0036; n = 4, Figure 4E).

### Effects of RO-10-5824 and surgery on slow γ band power

**Figure 5:**
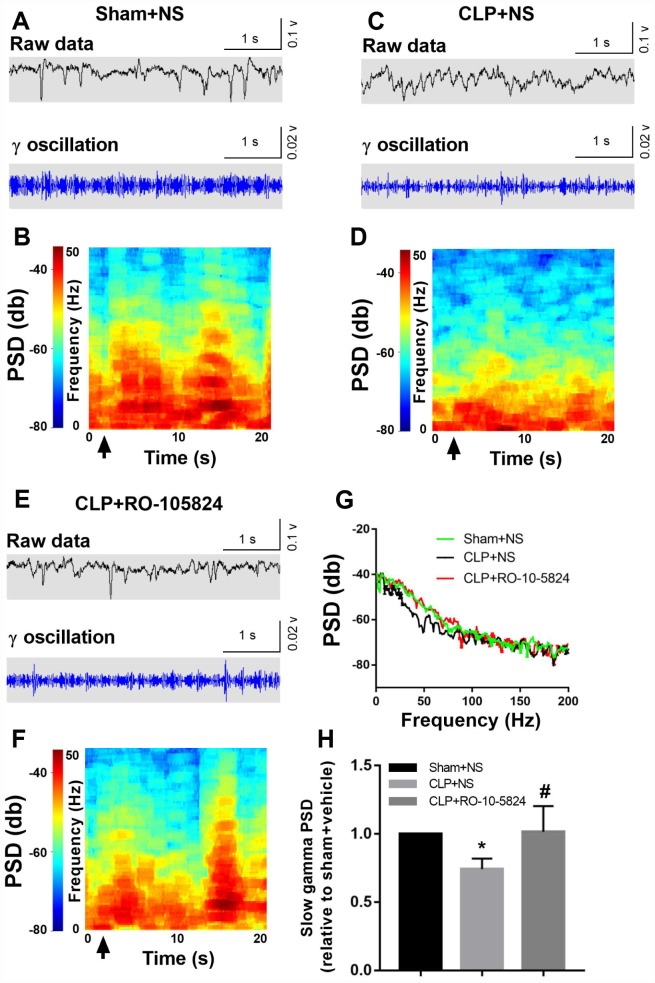
**RO-10-5824 treatment reversed CLP-induced decreased slow γ band power.** (**A**–**F**) Example recordings and example power spectra of γ oscillations in the hippocampal area CA1 of sham, CLP + NS, and CLP + RO-10-5824 groups. (**G**–**H**) Summary of LFP power. The slow γ band power was significantly higher in CLP + RO-10-5824 group when compared with CLP + NS group. Data are shown as mean ± SD (n = 5), *P < 0.05 vs sham + NS group, ^#^*P* < 0.05 vs CLP + NS group. LFP, local field potentials; PSD, power spectral density.

Power spectral analysis showed that CLP did not induce a significantly increased slow γ band power (30–50 Hz) compared with sham + NS group 14 days after surgery. These results suggest that the reduced inhibitory connectivity onto pyramidal neurons leads to altered neuronal synchrony and dysfunctions at the hippocampal network level. However, the slow γ band power was significantly higher in CLP + RO-10-5824 group when compared with CLP + NS group (F_2,8_ = 4.886, *P* = 0.0411, n = 5, Figure 5).

### RO-10-5824 treatment reversed CLP–induced cognitive impairments

**Figure 6:**
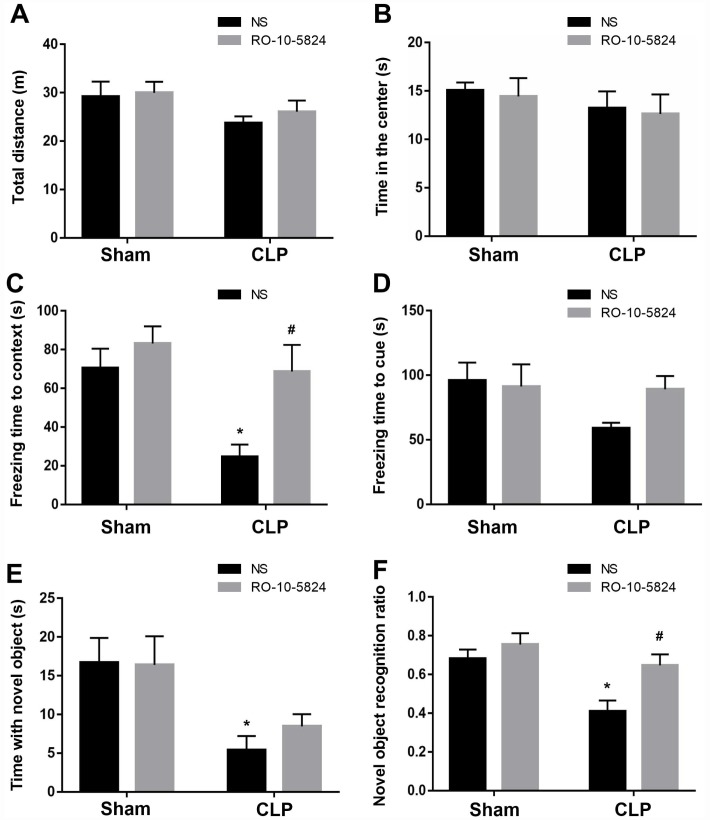
**RO-10-5824 treatment reversed CLP–induced cognitive impairment.** (**A**–**B**) CLP did not affect the distance traveled and time spent in center in the open arena. (**C**) CLP induced significantly decreased freezing time in the contextual fear conditioning, which was prevented by RO-10-5824. (**D**) There was no difference in the freezing time in the cued test. (**E**) CLP induced significantly decreased time with the novel object. (**F**) CLP induced significantly decreased novel object recognition ratio, which was reversed by RO-10-5824. Data are shown as mean ± SD (n = 11), *P < 0.05 vs sham + NS group, ^#^*P* < 0.05 vs CLP + NS group.

Behavioral tests were performed to assess cognition performance 14 days after surgery. As shown in Figure 6A, CLP did not affect the distance traveled in the open arena (F_1,40_ = 3.998, *P* = 0.054, n = 11). Also, there was no significant difference in the time spent in the center arena among the four groups (F_1,40_ = 1.194, *P* = 0.281, n = 11, Figure 6B), suggesting sepsis induced no anxiety-like behavior at 14 days after CLP. In the fear conditioning test, CLP induced significantly decreased freezing time in the contextual fear conditioning compared with sham NS group, whereas RO-10-5824 showed improved outcome (F_1,40_ = 8.929, *P* = 0.0048, n = 11), and there were no interaction effects (F_1,40_ = 2.419, *P* = 0.1278, n = 11, Figure 6C). In addition, there was no difference in the freezing time in the hippocampal-independent cued test among groups (F_1,40_ = 2.585, *P* = 0.1158, n = 11, Figure 6D). In the novel object recognition test, CLP induced significantly decreased time with the novel object (F_1,40_ = 12.53, *P* = 0.001, n = 11, Figure 6E) and novel object recognition ratio than those of sham + NS group, and there were no interaction effects, whereas RO-10-5824 treatment reversed the decreased novel object recognition ratio (F_1,40_ = 11.91, *P* = 0.0013, n = 11, Figure 6F).

## DISCUSSION

Brain dysfunction is not an uncommon complication in patients with sepsis and receives increasing attention [1–3]. In an animal study, it has been shown that sepsis induced memory and learning impairment 10 days but did not persist for 60 days after CLP [19]. Although locomotor activity was not affected, our study showed that sepsis induced cognitive impairments 14 days after CLP, as reflected by significantly decreased freezing time and novel object recognition ratio in the contextual fear conditioning and novel object recognition tests. Notably, one injection of D4 receptor agonist RO-10-5824 treatment was able to reverse decreased GABAergic transmission and γ oscillation, and cognitive impairments14 days after CLP. These results suggested that sepsis induced cognitive impairments probably by disrupting hippocampal PV interneuron-mediated inhibitory network via a D4-receptor mechanism.

Learning and memory retrieval are supported by information processing that requires a highly coordinated synchronous activity of neuronal assemblies modulated by local inhibitory microcircuits [20]. The brain is comprised of both excitatory neurons and inhibitory interneurons that work together to maintain the excitation/inhibition balance that is necessary for the normal functioning of the cortex [7, 21]. Indeed, it has been demonstrated that epileptiform discharges and electrographic seizures are more common in critically ill patients [22], suggesting excitation/inhibition balance alterations and glutamatergic and/or GABAergic transmission dysfunction. The PV interneurons are specific subtype of GABAergic neurons that comprise ~26% of the GABAergic neurons in the CA1 region, providing perisomatic inhibitory input onto pyramidal cells [9]. In the present study, we also showed a PV deficit in a sepsis model induced by CLP. Consistently, it has been shown that impaired PV interneuron function can be linked to network dysfunction and cognitive impairments in AD [23, 24]. Parallel to these data, dysregulation of PV interneuron was also observed in animal models of schizophrenia and depression [25, 26].

Perisomatic inhibition mediated by PV interneurons is pivotal in synchronizing the activity of pyramidal neurons and the generation of cortical γ oscillation that is essential for regulation of complex cognitive processes such as perception, memory, and attention [9, 10, 27]. By contrast, reduced γ oscillations and pyramidal synchronization has been observed in patients exhibiting symptoms of AD [28], whereas transplantation of inhibitory interneuron progenitors or prevention of GABAergic neuronal loss restored normal learning and memory in mice with Aβ accumulation [29]. In an animal model of AD, mice displayed reduced γ oscillations during exploratory activity [30], while moderate microglial inducible nitric oxide synthase-mediated nitic oxide release was sufficient to disturb γ oscillations in an in vitro study [31]. These findings raise the possibility that a PV deficit may result in disruption of inhibitory control in the brains of patients with sepsis, leading to deficits in learning and memory. In our study, we showed that sepsis also decreased CA1 γ oscillation, which is supported by one study demonstrating that deficit of PV interneurons and altered γ oscillation play a key role in working memory impairment in patients with schizophrenia [32].

Deficits in GABAergic inhibitory synaptic transmission may play a key role in impaired memory formation and consolidation [33]. In particular, PV interneurons are essential for modulating synchronizing activity and synaptic plasticity in the CA1 region, both of which are important for proper memory function [9]. There is mounting evidence suggesting impaired rhythmic spontaneous IPSCs originating specifically from PV interneurons in many psychiatric disorders. Reduced excitatory input onto interneurons was previously shown to cause disinhibition of pyramidal cells [34]. In addition, selectively targeted ablation of N-methyl-D-aspartate receptors selectively in PV interneurons induces abnormal hippocampal network synchrony and results in memory impairments [35]. In our study, we showed that sepsis induced a decrease in GABAergic synaptic output onto the pyramidal cell CA1 of the hippocampus, which was reflected by histological analyses and electrical recordings that directly assessed the monosynaptic connectivity between PV and pyramidal cells. This finding suggests that GABAergic output to the pyramidal neurons was impaired, probably due to a reduction in either number of GABAergic synapse or GABA release. Because the PV interneuron innervates the perisomatic domain of pyramidal neurons and plays a key role in generating network oscillations and cognition, we measured a special type of inhibitory synapse called the perisomatic synapse around the pyramidal neurons. We found that sepsis significantly decreased the total number of PV synapses around the NeuN-positive cells. Although NeuN is considered to be a pan-neuronal marker, one previous study suggested that NeuN-positive neurons with a typical pyramidal morphology were regarded as pyramidal neurons [36]. In addition, it has been shown that PV immunoreactivity in individual interneurons was closely correlated to GAD67 immunoreactivity under all experimental conditions [37]. These results indicated that perisomatic PV synapse can be considered as a marker of inhibitory synapse. Interestingly, RO-10-5824 treatment was unable to reverse the total number of PV synapses around the pyramidal neurons after CLP, suggesting RO-10-5824 increased GABAergic inhibitory transmission by promoting GABA release. Collectively, our study suggests that loss of PV inhibition by sepsis may result in decreased γ activity and contribute to the associated cognitive deficit. However, the cellular mechanism that induces abnormal γ oscillations following sepsis remains poorly understood.

Accumulating evidence has suggested the involvement of D4 receptors in cognitive functions by regulating the balance between glutamatergic excitation and GABAergic inhibition [38]. D4 receptors promote stabilization of neuronal functions by fine tuning synaptic plasticity based on the incoming information [39]. In particular, D4 receptors are enriched in GABAergic interneurons and modulate GABAergic transmission γ oscillations and potentially cognitive processes [40, 41]. Interestingly, it has also been demonstrated that D4 activation decreases excitatory synaptic strength in GABAergic interneurons and led to decreased GABAergic inhibition [42, 43]. The discrepancy likely depends on many factors including the brain area investigated and dose of D4 receptor agonist used. In our study, we showed that most PV interneurons co-express the D4 receptor and conversely a large proportion of D4 receptor-expressing neurons in the hippocampus co-express PV, which was significantly decreased after sepsis. Notably, Ro-10-5824, a highly selective D4 receptor agonist, was able to reverse γ power deficit and cognitive impairments. These results suggested that sepsis induced cognitive impairments by disrupting hippocampal PV interneuron-mediated inhibitory network through a D4-receptor mechanism.

In conclusion, our study suggests that sepsis induces cognitive impairments and this behavioral phenotype is accompanied by pronounced structural and functional changes affecting the integrity of hippocampal PV networks. In addition, we emphasize the value of the D4 receptor agonist for future clinical trials related to sepsis-induced cognitive impairments. However, it should be noted that the observed protection of the D4 receptor agonist in this model probably do not reflect specific effects on sepsis related changes of GABAergic neurotransmission because our chemical rescue strategy did not discriminate among neurons. Thus, using more specific method such as genetic manipulation is required to determine the precise role of D4 receptor in PV interneurons in our future research.

## MATERIALS AND METHODS

### Animals

Eighty-five Sprague–Dawley rats (male, 8 weeks old) were obtained from the Animal Center of Jinling Hospital, Nanjing, China, and were reared on a 12-h light–dark cycle (lights on at 07:00) at 24 ± 1°C. Standard rat chow and water were available *ad libitum* throughout the experiments. All animal protocols were approved by and performed in accordance with the Ethics Committee of Zhongda Hospital, Medical School, Southeast University, Nanjing, China, and the Guide for the Care and Use of Laboratory Animals from the National Institutes of Health (Bethesda, MD).

### Sepsis model

Sepsis model of CLP was performed as we previously described [7, 8]. Briefly, rats were anesthetized with phenobarbital sodium (*i.p.*, 40 mg/kg, Sigma-Aldrich), and a median laparotomy was made to expose the cecum under sterile surgical conditions, which was further ligated with 4-0 silk approximately 1 cm from the distal end. The cecum was then perforated twice with a sterile 22-gauge needle and was gently squeezed to extrude a small amount of fecal contents into the peritoneal cavity through the puncture site. The bowel was then situated back in the abdomen and the incision was sutured with a sterile 3-0 silk sutures. For the sham group, the abdominal cavity was opened without ligation or perforation. Immediately after the operation, all rats received fluid resuscitation with prewarmed (37 °C) normal saline solution (subcutaneously, 20 ml/kg of body weight) and antibiotic therapy (ertapenem, 20 mg/kg; Merck Research Laboratory, USA) and were then returned to their cages.

### Drug

RO-10-5824 was purchased from MedChemExpress (CAS NO: 189744-94-3). It was freshly dissolved in normal saline on the day of each experiment at a dose of 3 mg/kg. We choose this dose because previous study has demonstrated that it improves cognitive deficits [16]. In our present study, only one injection of RO-10-5824 was administered intraperitoneally (*i.p.*) 10 min before behavioral testing. Animals in the sham group received equivalent volumes (0.1 ml) of normal saline to control for injection stress.

### Neurobehavioral tests

Behavioral tests were performed as we described previously [7, 8]. All behavioral procedures were carried out between 8:00 and 12:00 a.m. in a sound–isolated room, with the instruments purchased from Shanghai Softmaze Information Technology Co., Ltd., China. Behavioral tests were conducted and results were recorded by two investigators who were blinded to the study protocol.

### Open field test

This test was carried out to evaluate the locomotor activity and anxiety behavior. The open field was a square plastic box of 100 cm × 100 cm × 40 cm and the ground of the open field was divided into 16 equal squares by red lines. Animals were left to freely explore it for 5 min and tracked by a photobeam activity system and software. The total distance traveled and time spent in the center of open arena was scored by an observer blinded to the animal grouping. At the end of each test, the arena was cleaned with 75% alcohol to eliminate olfactory cues.

### Novel object recognition test

This test was used to test memory and recognition using a plastic chamber (100 cm × 100 cm × 40 cm), which consisted of two trials. In the first day (training trial), the animals were placed in the box to explore two familiar objects for 5 min. In the probe test 24 h after the training trial, rats were placed in the same box, but one of the two familiar objects (5 cm × 5 cm × 5 cm) was exchanged with a novel object with a distinctively different color, shape, and texture and the rats were given 5 min to explore. The exploration time was recorded by a video camera and the novel object recognition ratio was calculated as follows: novel object recognition ratio = (time spent in novel object zone / time spent in novel object zone + time spent in familiar object zone. Object exploration was defined as a rat directed its nose toward an object at a distance of no more than 2 cm, and was actively investigating the object.

### Fear conditioning tests

Fear conditioning test was conducted by placing the rats into the conditioning chamber (32 cm × 25 cm × 25 cm). After 3 min of adaption, a single frequency sound signal (1 kHZ, 80 dB, 30 s, CS) was given to the rats. Then, an electric shock (1 mA, 2 s, US) was delivered through stainless steel bars. After 24 h, a context test to evaluate freezing behavior was performed by placing the rats in the same chamber again without any stimulation. The cued fear memory was tested 2 h later in a novel context with a continuous 3 min training tone presentation to monitor freezing behavior. Cognitive deficits was assessed by measuring the amount of time of freezing behavior, which was defined as the absence of all visible movement of the body except for respiration.

### In vivo electrophysiology

For LFP recording, rats underwent a chronic implant surgery as previously described [20]. Briefly, rats were anesthetized with phenobarbital sodium (*i.p.*, 40 mg/kg) and placed in a stereotaxic frame with precision micromanipulators. After craniotomy and removal of dura, a four-channel linear silicon probes were used to record unilateral CA1 region of the hippocampus. The coordinates were determined according to the rat brain atlas in stereotaxic coordinates (anterior, 3.8 mm; lateral, 1.8–2.2 mm; horizontal, 3.0 mm from bregma). To minimize the animals used, LFP was recorded only in sham + NS, CLP + NS, and CLP + RO-10-5824 groups. Local field potentials were recorded while the rats explored in the novel object recognition test. In our study, the bands were divided as follows, theta (3–8 Hz), α (8-15 Hz), β (15-30), low gamma (30–50 Hz) and fast gamma (50–100 Hz). The signals were filtered with a pass-band of 0.3–300 Hz and were further amplified and digitized at 2 kHz. The recorded LFPs were filtered by a 50 Hz notching filter to remove the powerline artifact. For LFP analysis, the wideband recordings were down-sampled at 1250 Hz. All data analyses were performed by Neuroexplorer (Plexon Inc., Dallas, TX) software.

### Whole-cell patch-clamp recording

Fourteen days after surgery, rats were anesthetized with phenobarbital sodium (*i.p.*, 60 mg/kg) and then decapitated. Coronal brain slices (350 μm thick) were cut with a VT 1000S microtome (Leica, Deerfield, IL) and was placed into sucrose-based solution containing in mM: 254 sucrose, 10 D-glucose, 3 KCl, 26 NaHCO_3_, 2 MgSO_4_, 2CaCl_2_, and 1.25 NaH2PO_4_, saturated with 95% O_2_/5% CO_2_, at pH 7.4, 300 mOsm. Slices were then immediately transferred into a holding chamber and were incubated in a mixture of sucrose and artificial cerebrospinal fluid (ACSF) containing in mM: 128 NaCl, 26 NaHCO_3_, 3 KCl, 2CaCl_2_, 10 D-glucose, 2 MgSO_4_, and 1.25 NaH2PO_4_. For whole-cell recordings, slices were transferred to a submersion-type recording chamber where they were continuously perfused with oxygenated ACSF. CA1 pyramidal cells were visually with a microscope equipped with a 40 × water-immersion objective coupled with an infrared differential interference contrast camera system. We focused on the CA1 region because it is the major output within the hippocampal trisynaptic circuit and its impairment is related to cognitive dysfunction [13]. Miniature inhibitory postsynaptic currents (mIPSCs) were recorded using glass pipettes (3–5 MΩ) filled with internal solution containing (mM) 145 CsCl, 10 EGTA, 5 NaCl, 10 HEPES-CsOH, 4 MgATP, and 0.3 Na_2_GTP in the presence of tetrodotoxin (1 μM), 2-amino-5-phosphonovaleric acid (100 μM), and 6-cyano-7-nitroquinoxaline-2,3-dione (200 μM). A MultiClamp 700B amplifier and a Digidata 1440A A-D converter (Axon Instruments, Union City, CA) were used for data acquisition, and data were analyzed with pClamp10 (Axon Instruments).

### Western blotting

Proteins extracted from the hippocampus were processed for western blotting. Hippocampal samples were homogenized in ice-cold lysis buffer (1% Nonidet P-40, 0.1% sodium deoxycholate, 0.1% SDS, 66 mM EDTA, and 10 mM Tris-HCl, pH 7.4) supplemented with a protease inhibitor cocktail. After centrifuging at 13,000 g for 10 min at 4 °C., the supernatant was saved and the protein concentration was determined by Bradford assay. Equal amounts of sample (40 μg) were loaded per lane and electrophoresed on SDS-PAGE gels. The separated proteins were then transferred to polyvinylidene fluoride membranes. After the membranes were blocked with 5% skim milk in Tris-buffered saline with Tween (TBST), they were incubated with each primary antibody: polyclonal rabbit anti-PV (1:1000; Abcam, Cambridge, UK), monoclonal mouse anti-D4 (1:1000; Santa Cruz Biotechnology, Dallas, TX), and rabbit anti-GADPH (1:10000; Cell Signaling Technology, Boston, MA) overnight at 4 °C. temperature. After they were thoroughly washed, membranes were incubated in Tris-buffered saline tween with goat anti-rabbit and goat anti-mouse IgG-horseradish peroxidase-conjugated secondary antibodies (1:7000, Bioworld Technology, St. Louis Park, MN, USA) for 1 h at room temperature. The bands were detected with Pierce ECL Western Blotting Substrate (Thermo Fisher Scientific, Rockford, IL) and quantified with Image J software (National Institutes of Health, Bethesda, MD).

### Immunohistochemistry

The rats were deeply anesthetized with phenobarbital sodium (*i.p.*, 60 mg/kg) and perfused transcardially with normal saline, followed by 4% paraformaldehyde in phosphate buffered saline (PBS). The brains were harvested and postfixed in 4% paraformaldehyde, and then dehydrated in 30% sucrose overnight at 4 °C.. Samples were embedded in Optimal Cutting Temperature compound, cut into 30 μm thick sections using a cryostat, and mounted on slides (Leica, Nussloch, Germany). Slices were blocked with 3% bovine serum albumin for 1 h at room temperature. We used rabbit anti-PV (1:500; Abcam, Cambridge, UK), rabbit anti-D4 (1:500; Proteintech, Chicago, IL), and mouse anti-NeuN (1:1000; Proteintech, Chicago, IL) as primary antibodies. After the sections were washed three times with PBS, they were incubated with appropriate secondary antibodies, including goat anti-rabbit and mouse IgG-FITC or -Cy3 (1:300; Santa Cruz Biotechnology 1:500, Life Science Technology) for 1 h at room temperature. After the secondary antibody was washed out, the sections were incubated with 4′, 6-diamidino-2-phenyl-indole (DAPI) for nuclear staining. Image J was used to measure the mean value of the immunofluorescence in each section.

For analysis of perisomatic PV synapse formation, the z-stack high-magnification colocalization images for NeuN and PV were obtained. The slices were imaged every 1 μm across the entire slice and then collapsed together to form one image of the resulting z-stack picture. The total number of PV synapses was counted in 3 to 5 fields/section in 3 to 5 sections/brain as previously described [36]. All images were acquired using the same parameters, and PV synapses were counted using NIH ImageJ software.

### Statistical analysis

Statistical analysis was performed using GraphPad Prism 7.0 (GraphPad Software, La Jolla, CA, USA). Data are presented as mean ± SD. All presented data were evaluated for normal distribution by Kolmogorov–Smirnov test. Student’s t-test or Mann-Whitney test was used to assess differences between two groups according to the distribution of the data. Differences among multiple groups were tested using a one-way analysis of variance test for normal distribution data or a nonparametric Kruskal–Wallis test for a non-normal distribution where appropriate. For the behavioral data, two-way analysis of variance was used, followed by Bonferroni post-hoc test. The survival rate was estimated by the Kaplan–Meier method and compared by the log–rank test. *P*-value < 0.05 was considered significant.
